# kataegis: an R package for identification and visualization of the genomic localized hypermutation regions using high-throughput sequencing

**DOI:** 10.1186/s12864-021-07696-x

**Published:** 2021-06-12

**Authors:** Xue Lin, Yingying Hua, Shuanglin Gu, Li Lv, Xingyu Li, Pin Chen, Peng Dai, Yunyun Hu, Anna Liu, Jian Li

**Affiliations:** 1 Department of Bioinformatics, School of Biomedical Engineering and Informatics, Nanjing Medical University, 211166 Nanjing, People’s Republic of China; 2 Traditional Chinese Medicine Department, Fuxing Hospital, Capital Medical University, 100038 Beijing, People’s Republic of China; 3 Key Laboratory of DGHD, MOE, School of Life Science and Technology, Southeast University, 210096 Nanjing, People’s Republic of China

**Keywords:** Kataegis, Visualization, High-throughput sequencing, R

## Abstract

**Background:**

Genomic localized hypermutation regions were found in cancers, which were reported to be related to the prognosis of cancers. This genomic localized hypermutation is quite different from the usual somatic mutations in the frequency of occurrence and genomic density. It is like a mutations “violent storm”, which is just what the Greek word “kataegis” means.

**Results:**

There are needs for a light-weighted and simple-to-use toolkit to identify and visualize the localized hypermutation regions in genome. Thus we developed the R package “kataegis” to meet these needs. The package used only three steps to identify the genomic hypermutation regions, i.e., i) read in the variation files in standard formats; ii) calculate the inter-mutational distances; iii) identify the hypermutation regions with appropriate parameters, and finally one step to visualize the nucleotide contents and spectra of both the foci and flanking regions, and the genomic landscape of these regions.

**Conclusions:**

The kataegis package is available on Bionconductor/Github (https://github.com/flosalbizziae/kataegis), which provides a light-weighted and simple-to-use toolkit for quickly identifying and visualizing the genomic hypermuation regions.

## Background

There are numerous somatic mutations in human genomes, especially in cancer genomes. Many exogenous and endogenous factors are known reasons for the occurrence of the somatic mutations, like the ultra-violet lights, chemical mutagens, and DNA repair, etc. [1] And different mutational combinations are usually generated by different mutational processes, e.g., C > T and CC > TT transitions are common in ultra-violet light related skin cancers [2], and G > T in aflatoxin-B1 associated hepatocellular carcinomas [3]. The mutational combinations are called “signatures” of the mutational processes. These signatures were firstly analyzed in a small number of frequently mutated cancer genes like the TP53, however with the rapid development of the massively parallel sequencing technology, it has overcome the scale limitations, thus tens of thousand of variations can be identified in a cancer genome. Intriguingly, when screening the mutations and extracting the signatures, the researchers investigated a possibility of regional clustering of mutations by constructing “rainfall plots”, which present the inter-mutational distances between each mutation [4]. These regional clustering mutations are “hotspots” of mutations in caner genomes, i.e., regional hypermutation, which is just like a “violent storm” of the mutations in the cancer genomes, thus this phenomena is named with a Greek word “kataegis” which exactly means the same.

The kataegic foci were earlier investigated in a study of 21 breast cancers [4, 5]. Later with 7,042 primary cancers of 30 different classes analyzed, cancers of breast (67 of 119), pancreas (11 of 15), lung (20 of 24), liver (15 of 88), medulloblastomas (2 of 100), CLL (Chronic Lymphocytic Leukemia) (15 of 28), B-cell lymphomas (21 of 24) and acute lymphoblastic leukaemia (1 of 1) showed occasional (< 10), small (< 20 mutations) foci of kataegis [6]. The mechanism of the generation of the kataegic foci is not fully clear. But the kataegis foci were usually found co-locating with the genomic rearrangements. There was evidence in yeast and human that the clustered mutations can arise from damaged long single-strand DNA regions [7], the chromothripsis and kataegis were induced by telomere crisis [8], and the AID/APOBEC editing deaminases were involved [9–11]. Further analysis of the kataegis expression signature found that, in breast cancer it is associated with late onset, better prognosis and higher HER2 levels [12]. To decipher the role of the kataegis in the cancer genomes, there are needs for a light-weighted and simple-to-use toolkit to identify and visualize the localized hypermutation regions in genome. Thus we developed the R package “kataegis” to meet these needs.

## Implementation

This package was coded in R language with RStudio version 1.2.5042 built on R version 4.0 on macOS Mojave version 10.14. It depends on the R core packages grDevices, graphics, stats, and utils, and is maintained and released through the Bioconductor project with an Artistic License.

**Fig. 1:**
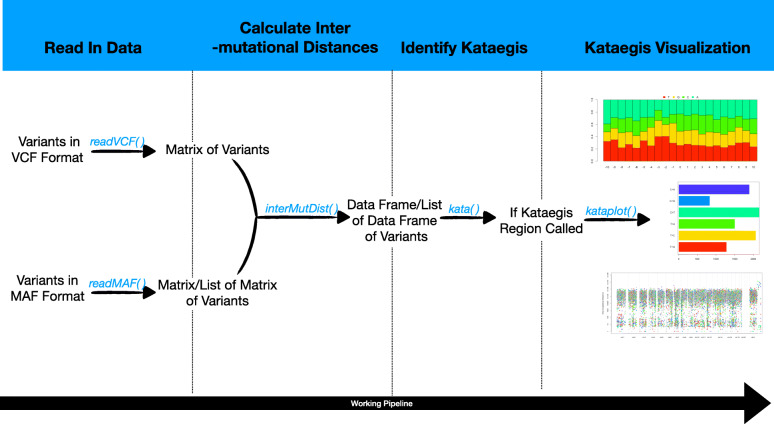
The overview of the working flow of the kataegis package

The kataegis package provides a four-step workflow for localized hypermutation regions identification and visualization: data read in, inter-mutational distances calculation, kataegis identification, and visualization (Fig. 1).

The readVCF() and readMAF() functions can read in the standard Variant Call Format (VCF) (http://github.com/samtools/hts-specs/blob/master/VCFv4.2.pdf) and Mutation Annotation Format (MAF) (http://docs.gdc.cancer.gov/Data/File_Formats/MAF_Format/) files respectively. The VCF and MAF formats are both most commonly used file formats for storing variants information from high-throughput sequencing, e.g., whole genome sequencing. Both of these formats have standard specifications, and there are also mature tools to perform filtering and format conversion between them. The readVCF() function will read in the VCF format and suffixed files and do a crude filtering according to the VCF “FILTER” field. As the MAF file can hold the mutations’ annotation data of several samples, which is widely used by important bioinformatic databases like the The Cancer Genome Atlas (TCGA), etc., so we provide a function readMAF() to read in the MAF format and suffixed files with the samples merged or separated. If the user chooses to read in the MAF file with the samples separated, then the variants will be read in to a list of matrix, each matrix is named after the sample’s ID. The crude filtering also works for the readMAF() function. The package contains two simulated data sets, of which the VCF was generated form the mouse model of small cell lung cancer GSE149444_17686R (http://www.ncbi.nlm.nih.gov/geo/query/acc.cgi?acc=GSE149444) and the MAF was generated from the human adenoid cystic carcinoma (http://www.cbioportal.org/study/summary?id=acyc_mskcc_2013) [13].

If the data is read in without any errors, the second step is to calculate the inter-mutational distances between the mutations. The variants will firstly be sorted according to the genomic coordinates for each chromosome, and then the distances will be calculated between the neighboring variants. For a list of separated samples, the samples with too few variants for calculating the inter-mutational distances will be abandoned. And a warning of this situation and the sample IDs will arise. This step will produce a data frame containing the information of the chromosomes, variants’ locations, and the inter-mutational distances.

With the previous two steps, the data is ready for calling the localized hypermutation regions. The localized hypermutation regions are mutations “hotspot” regions, which are defined as more than a certain number of mutations in a range of the genome. It was reported as more than five [9] or six mutations in a range of 1000 bp of the human genome [6]. With this concept, it is reasonable to segment the genomes with the mutations. We used a segmentation method based on the Piecewise Constant Fitting (PCF) algorithm [14]. The segmentations with the information of the number of mutations and its average inter-mutational distances are reported, and the genomic coordinates are also produced. Thus it’s simple for users to filter the segments with the threshold of the mutation number and the average inter-mutational distance. The kata() function will automatically perform the previous jobs.

**Fig. 2:**
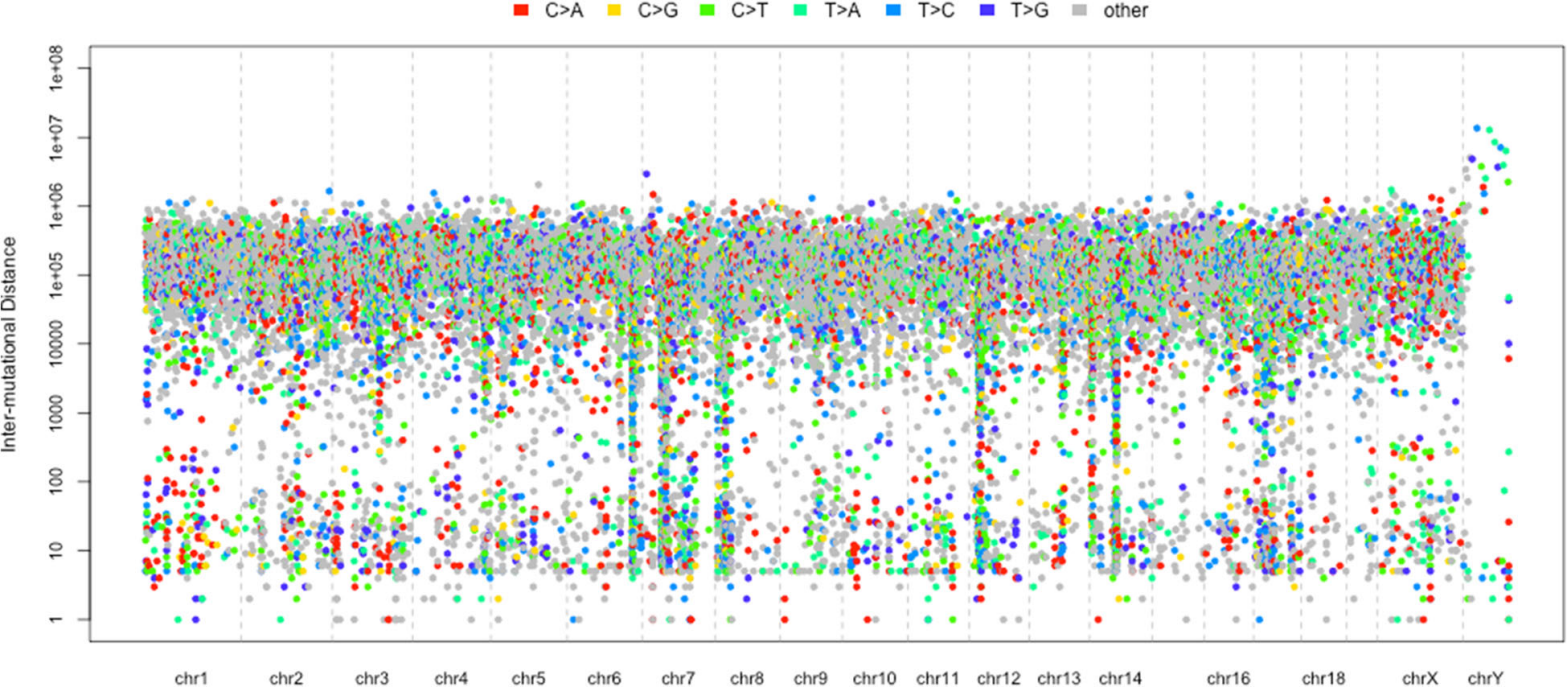
The rainfall plot of all the mutations across the genome. The x-axis presents the chromosome positions and the y-axis presents the inter-mutational distances. The different colors present the different types of the mutation substitutions. The clustered mutations of both chromosome positions and the inter-mutational distances may present the kataegis

**Fig. 3:**
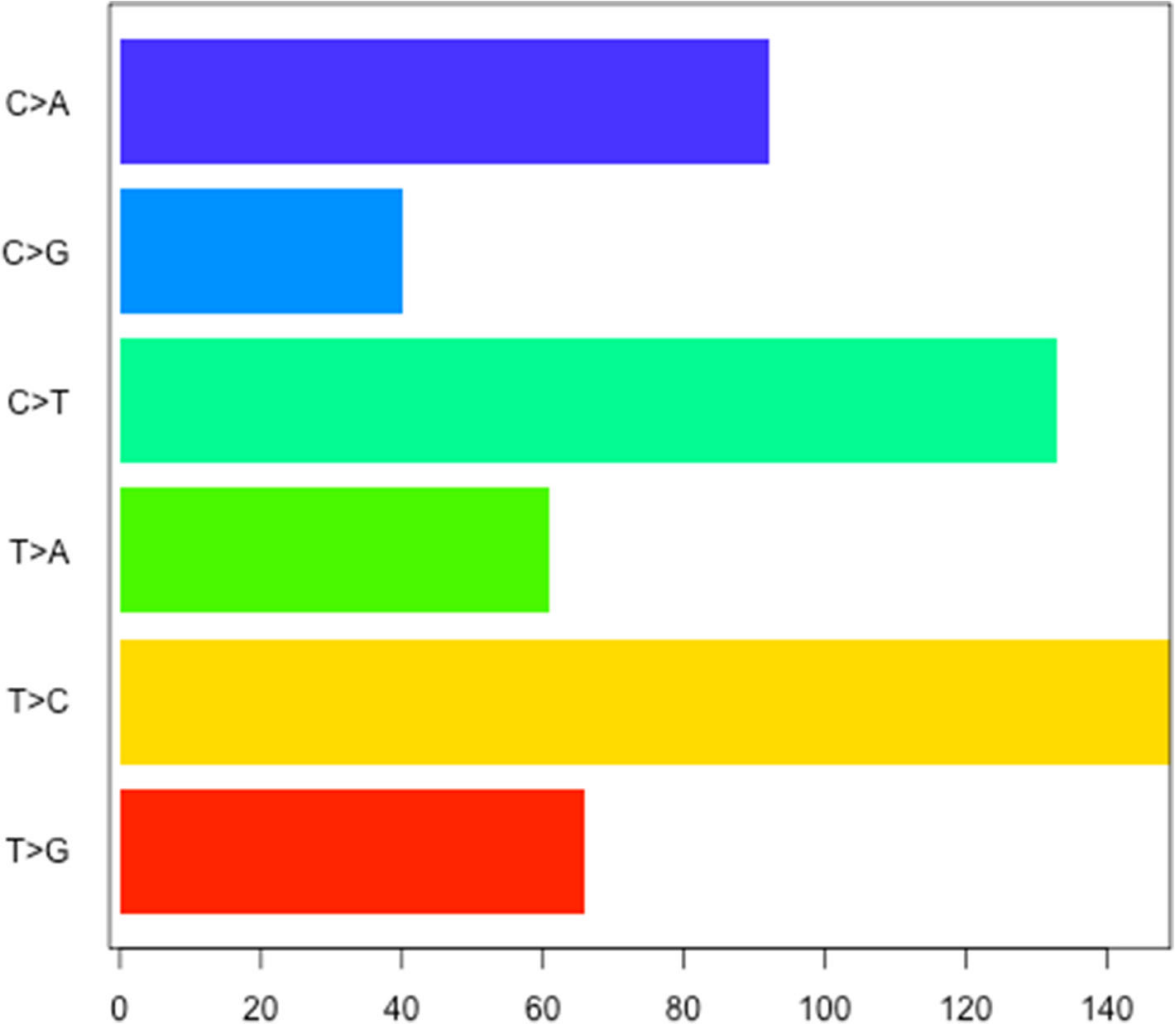
The horizontal bar plot of the mutation substitution types of the kataegic foci

**Fig. 4:**
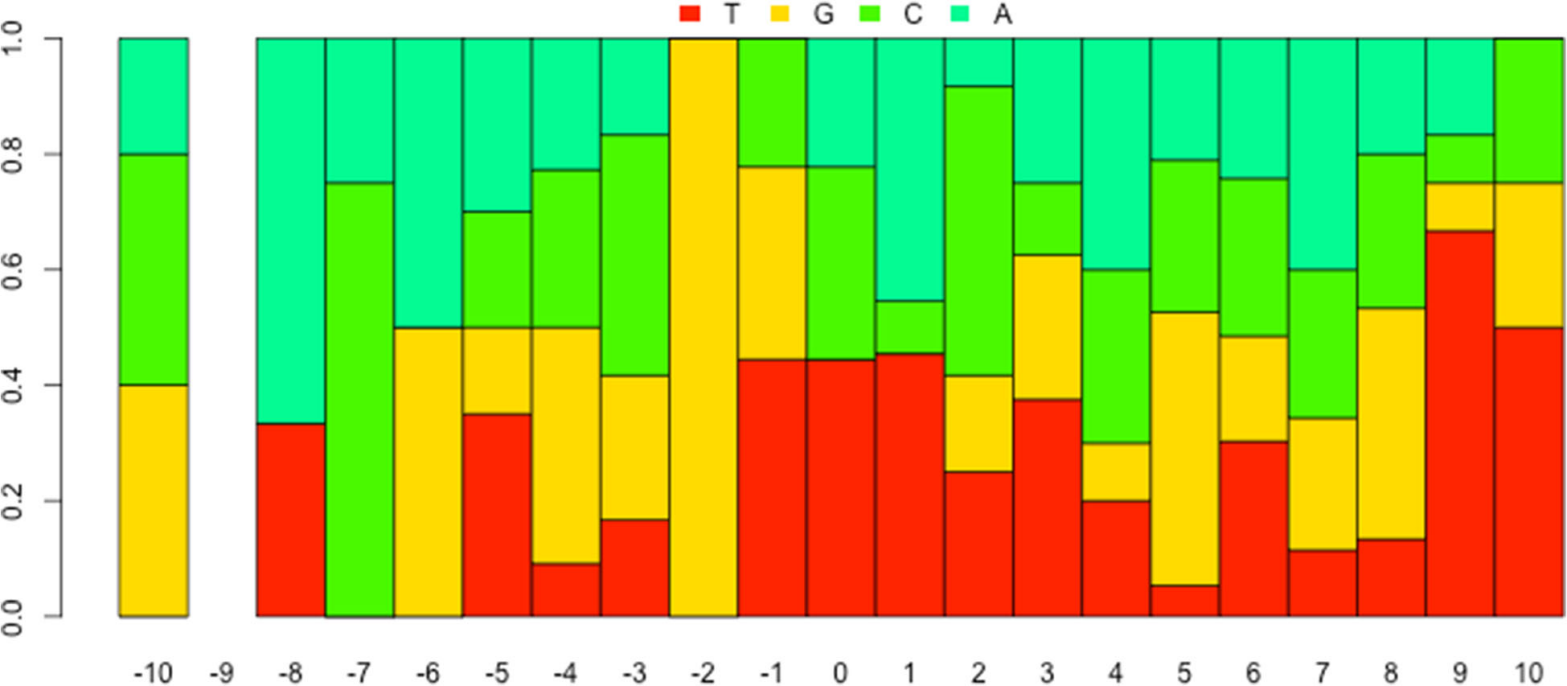
The stack bar plot of the nucleotide content of the kataegic foci and flanking region

**Fig. 5:**
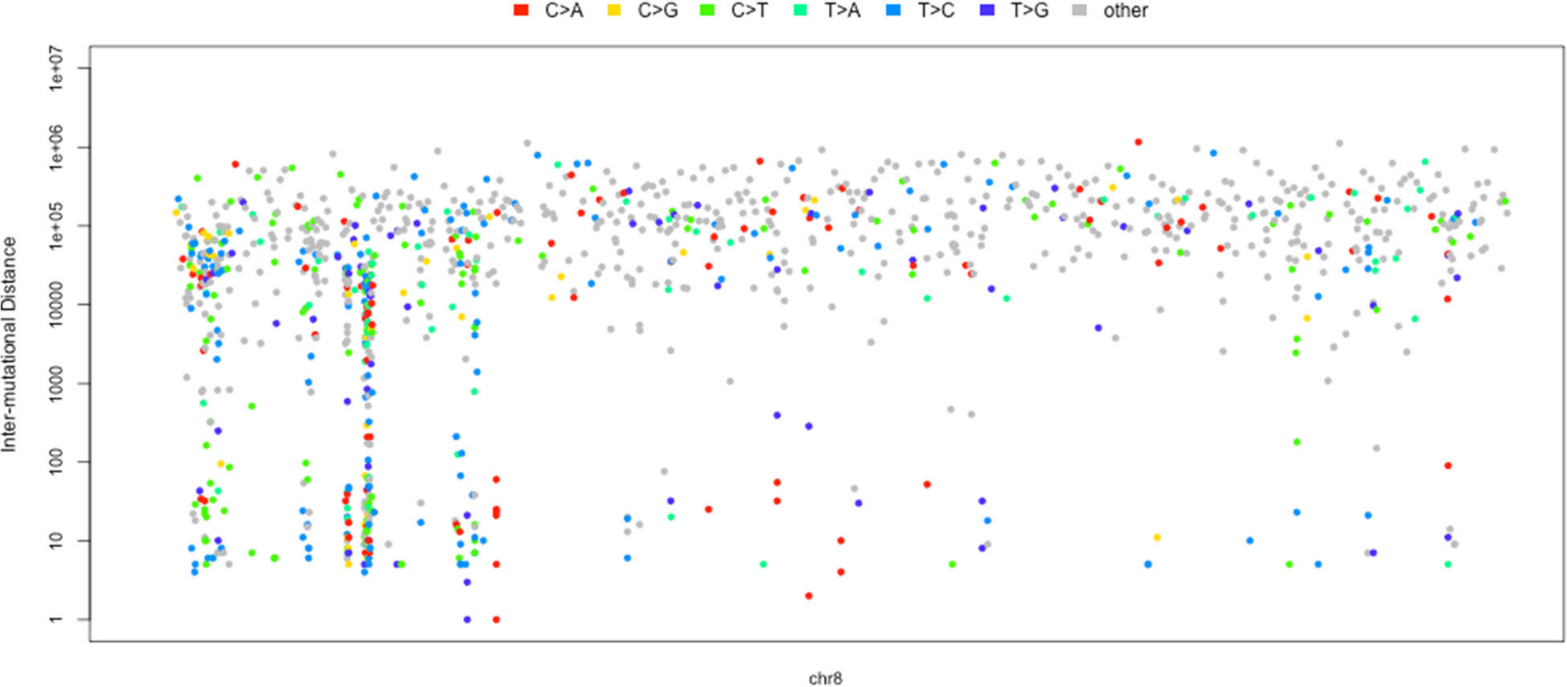
The rainfall plot of all the mutations of one chromosome: chromosome 8

After all the first three steps finished, and the localized hypermutation regions are identified successfully, it comes to the last step to visualize the regions. Researchers are usually interested in the global landscape of the distribution of the regions in the genome (Fig. 2), and also the nucleotides content (Fig. 3) and spectra (Fig. 4) of the foci and flanking regions of the localized hypermuation regions. Here we provide a kataplot() function, it will take in the data produced by the first three steps and produce these plots automatically. The users only have to control which type of plots will be produced, the size and format of the plots, and the name of the plots as well. The global landscape of the distribution of the regions can also become the landscape of one or several certain chromosomes other than the whole genome (Fig. 5).

## Results and discussion

We have prepared a set of simulated VCF files by using the script coded by ourselves, which is available on https://github.com/flosalbizziae/BioinfoCollects/blob/main/katasim.py. The script can mutate the DNA sequence and provide the users an option –k to control whether or not to form kataegis during the mutating of the sequence. When the –k option is set to 1, the script will form kataegis in the sequence by mutating more than 6 mutations within 1 kb, which is just the same for defining the kataegis, and the kataegis will be randomly distributed on the sequence. When the –k is set to 0, the script will mutate the sequence completely randomly, and at the same time ensure that the mutations are not too close to each other to trigger the definition of kataegis. With this script, a hundred VCF files were generated, giving the same tumor mutational burden (TMB) on the same sequence, the chr1 of human genome version hg38 downloaded from UCSC Genome Browser web site. One half of the simulated VCFs are with kataegis, and the other half are without kataegis. Analyzing with our package, we obtained good sensitivity and specificity. We detected kataegis from all the fifty VCFs generated with kataegis, and on the other hand, we detected no kataegis from all the other fifty VCFs generated without kataegis. The sensitivity of the detection of kataegis largely depends on the quality of the definition of the mutations. As the package will segment the sequence according to the mutations and the inter-mutational distances, if the mutations were not detected in the variants calling analysis, then kataegis cannot be detected consequently. Thus our stringent criteria of the segmentation may under-represent the kataegis in the analysis.

## Conclusions

In conclusion, we have provided a light-weighted, simple-to-use, and relatively flexible toolkit for identifying and visualizing the localized hypermutation regions using high-throughput sequencing data as an R package. This toolkit uses a straight-forward and statistical strategy to identify the kataegis regions, which provides the users a convenient usage experience and efficient researching tool for variants understanding and further study of integrating the mutations with the clinical outcomes in a new dimension.

## Availability and requirements

Project name: kataegis.

Project page: https://github.com/flosalbizziae/kataegis.

Operating systems: platform independent.

Programming language: R.

Other requirements: R 4.0 or higher.

License: Artistic-2.0.

Any restriction to use by non-academics: Artistic-2.0 license needed.

## Data Availability

The system data for building the package, including hg17, hg18, hg19, hg38, mm7, mm8, mm9, and mm10, was downloaded from the UCSC genome browser using its tool Table browser (https://genome.ucsc.edu/cgi-bin/hgTables?hgsid=1097046771_lLSM8fFqrnzE9fECLP2zBMeNfPZ6) with the options group setting to “Mapping and Sequencing” and track setting to “Chromosome Band (Ideogram)”. The VCF example file was generated from the GEO data set GSE149444 (https://www.ncbi.nlm.nih.gov/geo/query/acc.cgi?acc=GSE149444), and the MAF example file was generated from the human adenoid cystic carcinoma data of TCGA database, which was downloaded from http://www.cbioportal.org/study/summary?id=acyc_mskcc_2013. The VCF files for simulation was generated from the hg38 genome data, which was downloaded from the UCSC genome browser using its tool Downloads (https://hgdownload.soe.ucsc.edu/goldenPath/hg38/chromosomes/).

## Abbreviations

CLL: Chronic Lymphocytic Leukemia
VCF: Variant Call Format
MAF: Mutation Annotation Format
TCGA: The Cancer Genome Atlas
PCF: Piecewise Constant Fitting
TMB: Tumor Mutational Burden
